# Nicotine induces EP4 receptor expression in lung carcinoma cells by acting on AP-2α: The intersection between cholinergic and prostanoid signaling

**DOI:** 10.18632/oncotarget.18023

**Published:** 2017-05-19

**Authors:** Yu Fan, Ke Wang

**Affiliations:** ^1^ Department of Radiotherapy, Sichuan Cancer Hospital and Institute, Sichuan Cancer Center, School of Medicine, University of Electronic Science and Technology of China, Chengdu, P.R. China 610041; ^2^ Department of Respiratory Medicine, West China Hospital, Sichuan University, Chengdu, Sichuan Province, P.R. China 610041; ^3^ Lung Cancer Centre, West China Hospital, Sichuan University, Chengdu, Sichuan Province, P.R. China 610041

**Keywords:** nicotine, non-small cell lung carcinoma, acetylcholine receptor, cyclooxygenases-2, proliferation

## Abstract

It was demonstrated that nicotine increased non-small cell lung cancer cell proliferation through nicotinic acetylcholine receptor -mediated signals. However, the detailed mechanism remains incompletely understood. We evaluated whether nicotine increased EP4 receptor expression in lung carcinoma cells by activating on AP-2α. Methods: The non-small cell lung cancer cells of A549 and H1838 were cultured and treated with EP4 inhibitor AH23848, also with EP4 and control siRNAs. The extracellular signal-regulated kinases inhibitor PD98059, the p38 mitogen-activated protein kinase inhibitor SB239063, the α7 nicotinic acetylcholine receptor inhibitor α-bungarotoxin, the α4 nicotinic acetylcholine receptor inhibitor dihydro-β-erythroidine, the PI3K inhibitor wortmannin, the PKC inhibitor calphostin C, and the PKA inhibitor H89 have been used to evaluate the effects on proliferations. It indicates that nicotine increases EP4 expression through α7 nicotinic acetylcholine receptor-dependent activations of PI3-K, JNK and PKC pathways that leads to reduction of AP-2α-DNA binding. This, together with the elevated secretion of PGE_2_, further enhances the tumor promoting effects of nicotine. These studies suggest a novel molecular mechanism by which nicotine increases non-small cell lung cancer cell proliferation.

## INTRODUCTION

Tobacco exposure is one of the most important risk factors for the development of lung carcinoma and causes more than 440,000 deaths annually in United States ^1^. In particular, NSCLC shows a strong etiologic association with smoking. Nicotine, a major alkaloid present in tobacco, has been shown to induce cancer cell proliferation and angiogenesis and to inhibit apoptosis through specific nicotinic acetylcholine receptors (nAChRs) [2, 3]. However, the molecular mechanisms underlying the role that nicotine plays in promoting lung cancer progression remain incompletely understood.

Cyclooxygenases-2 (COX-2) is expressed in a high percentage of premalignant lesions and established tumors. One of the bioactive products of COX-2, prostaglandin E_2_ (PGE_2_), has been implicated in a variety of biological processes including cell proliferation, tissue invasion, apoptosis, and angiogenesis [4]. The effect of PGE_2_ has been attributed to its known capacity to bind to its prostanoid receptors designated EP1, EP2, EP3 and EP4 [5]. Of these, EP4 has received much attention because it is overexpressed in many cancer cells including lung carcinoma cells, and it has been found to be involved in promoting the growth and invasion of NSCLC in many experimental systems [6] .

Since both cholinergic and prostanoid signaling appear to be key drivers of tumor progression, we directed our attention to investigating the potential link between these pathways [7, 8]. We found that nicotine stimulation of lung carcinoma cell growth was partly dependent upon PGE_2_ production. Furthermore, nicotine also stimulated the expression of EP4 and this effect was mediated via activation of several kinase pathways including PI3-K, JNK and PKC, which, in turn, affected the transcription factors AP-2 resulting in upregulation of EP4 expression. Together, these data suggest that cholinergic signaling through nAChRs stimulates prostanoid signaling through the induction of PGE_2_ and EP4 expression thereby revealing a mechanistic link between these two pro-oncogenic pathways.

## RESULTS

### Nicotine-induced lung cancer cell growth is, in part, dependent on EP4

**Figure 1:**
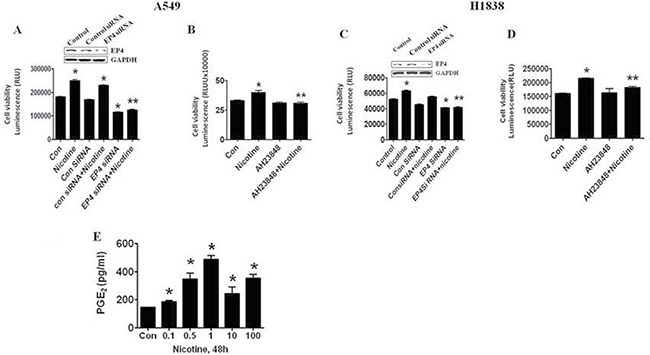
Nicotine stimulates lung cancer cell growth through induction of EP4 (**A**) EP4 SiRNA decreased the proliferation of A549 cells induced by nicotine (0.5 μM). (**B**) AH23848 decreased the proliferation of A549 cells induced by nicotine (0.5 μM). (**C**) EP4 SiRNA decreased the proliferation of H1838 cells induced by nicotine (0.5 μM). (**D**) AH23848 decreased the proliferation of H1838 cells induced by nicotine (0.5 μM). (**E**) Nicotine increased secretion of PGE_2_ in dose-dependent manner in A549 cells in ELISA assay. ^*^indicates significantly difference from control. ^**^indicates significance of combination treatment as compared with nicotine alone (*P* < 0.05). *Con* indicates untreated control cells.

While exploring the mechanisms by which nicotine affects carcinoma cell proliferation, we found that blockade of EP4 influenced this process. We silenced the *EP4* gene in cultured cells using siRNA approaches. A549 cells transfected with EP4 siRNA duplexes were plated in DMEM with 0.5% FBS for 48 h containing 0.5 μM nicotine for an additional 72 h. As shown in Figure 1A, knockdown of the *EP4* gene inhibited nicotine-induced A549 cell proliferation as determined by cell viability assays. Note that silencing of EP4 largely reduced EP4 protein expression (Figure 1A upper panel, 0.783 ± 0.106 vs 1.000 ± 0.046, *P* < 0.01) and the control siRNA had no effects (0.993 ± 0.048vs 1.000 ± 0.046, *P* > 0.05). As expected, a specific EP4 inhibitor, AH23848, also inhibited the effect of nicotine-induced A549 cell proliferation (Figure 1B). Similar results were also found in an additional NSCLC cell line (H1838) (Figure 1C upper panel, EP4 siRNA 0.819 ± 0.073 vs 1.000 ± 0.039, *P* < 0.01; control siRNA 0.999 ± 0.020 vs 1.000 ± 0.039, *P* > 0.05; Figure 1D).

The above results suggested that nicotine acts, at least in part, through prostanoid receptors. We predicted that these effects would be mediated by the indirect induction of PGE_2_ release by nicotine. This was confirmed by measuring PGE_2_ levels in the supernatants of cells exposed to nicotine (Figure 1E).

### Nicotine stimulated the expression of EP4

**Figure 2:**
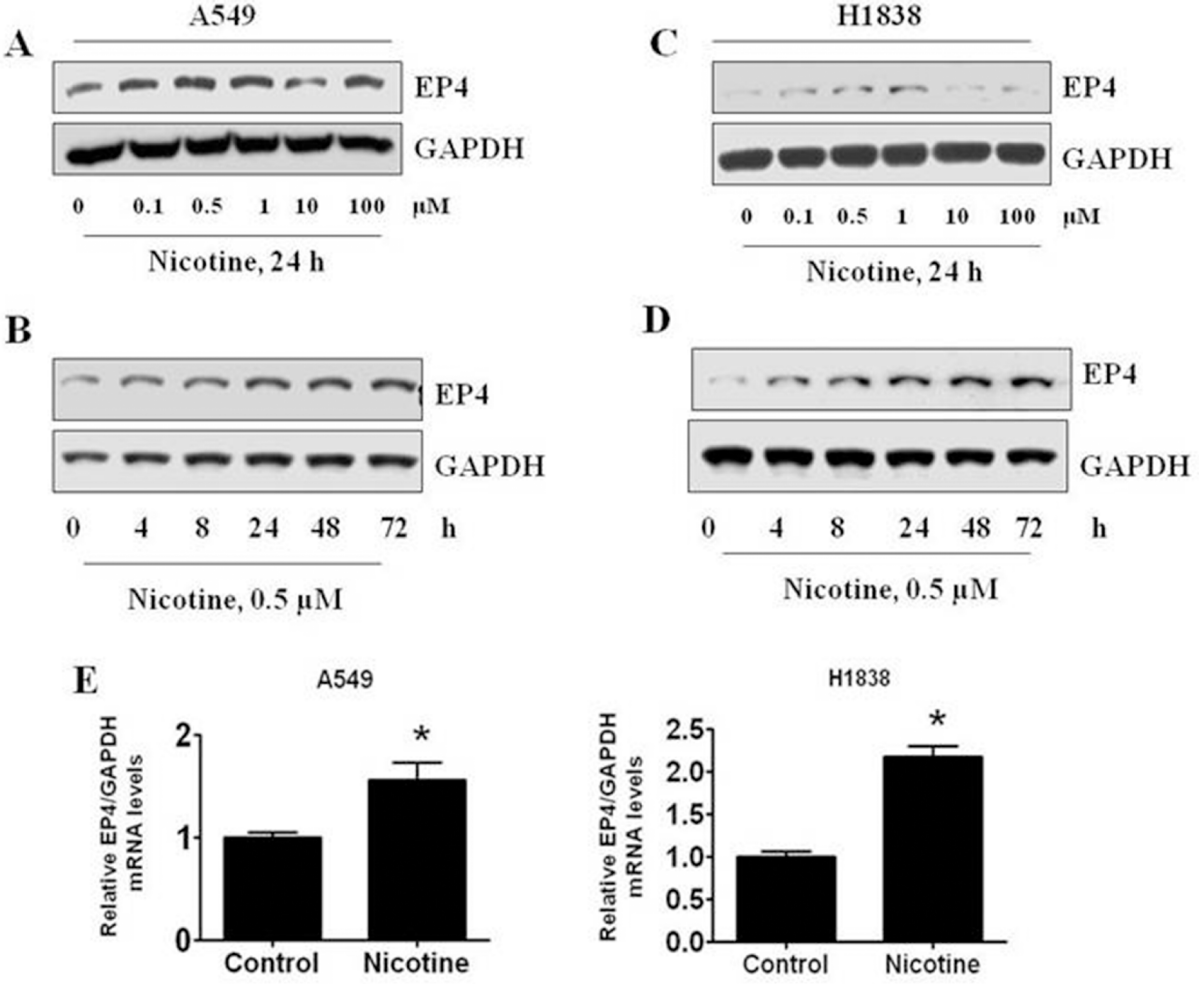
The effects of nicotine, acetylcholine, and acetylcholinesterase on EP4 expression in human lung carcinoma cells (**A**) Nicotine increased the expression of EP4 in dose-dependent manner in A549 cells. (**B**) Nicotine increased the expression of EP4 in time-dependent manner in A549 cells. (**C**) Nicotine increased the expression of EP4 in dose-dependent manner in H1838 cells. (**D**) Nicotine increased the expression of EP4 in time-dependent manner in H1838 cells. E. Nicotine increased EP4 mRNA expression as determined by real time RT-PCR. GAPDH served as internal control for normalization purposes. ^*^indicates significant differences from control (*P* < 0.05).

We also found that nicotine stimulated EP4 gene expression. A549 NSCLC cells exposed to nicotine showed increased EP4 protein levels in a time- and dose-dependent manner with maximal increases noted at a concentration of 0.5 μM at 24h (Figure 2A, 1.307 ± 0.143 vs 1.009 ± 0.023, *P* < 0.01; Figure 2B 1.249 ± 0.198 vs 1.002 ± 0.015, *P* < 0.01). Similar results were also observed in H1838 cells (Figure 2C, 1.164 ± 0.089 vs 1.011 ± 0.017, *P* < 0.05; Figure 2D 1.333 ± 0.126 vs 1.007 ± 0.021, *P* < 0.01). Nicotine also significantly increased EP4 mRNA levels as determined by real-time RT-PCR in A549 and H1838 cells (Figure 2E).

Together, these results suggested that nicotine stimulates lung carcinoma cell proliferation through the release of PGE_2_ which, in turn, acts on EP4 receptors to promote proliferation. Importantly, this effect might be amplified by the ability of nicotine to stimulate the expression of EP4. Considering the importance of this effect, we proceeded to evaluate the mechanisms by which nicotine stimulates EP4 expression.

### α7 nAChR and PI3-K, JNK and PKC signaling are involved in nicotine-induced EP4 expression

**Figure 3:**
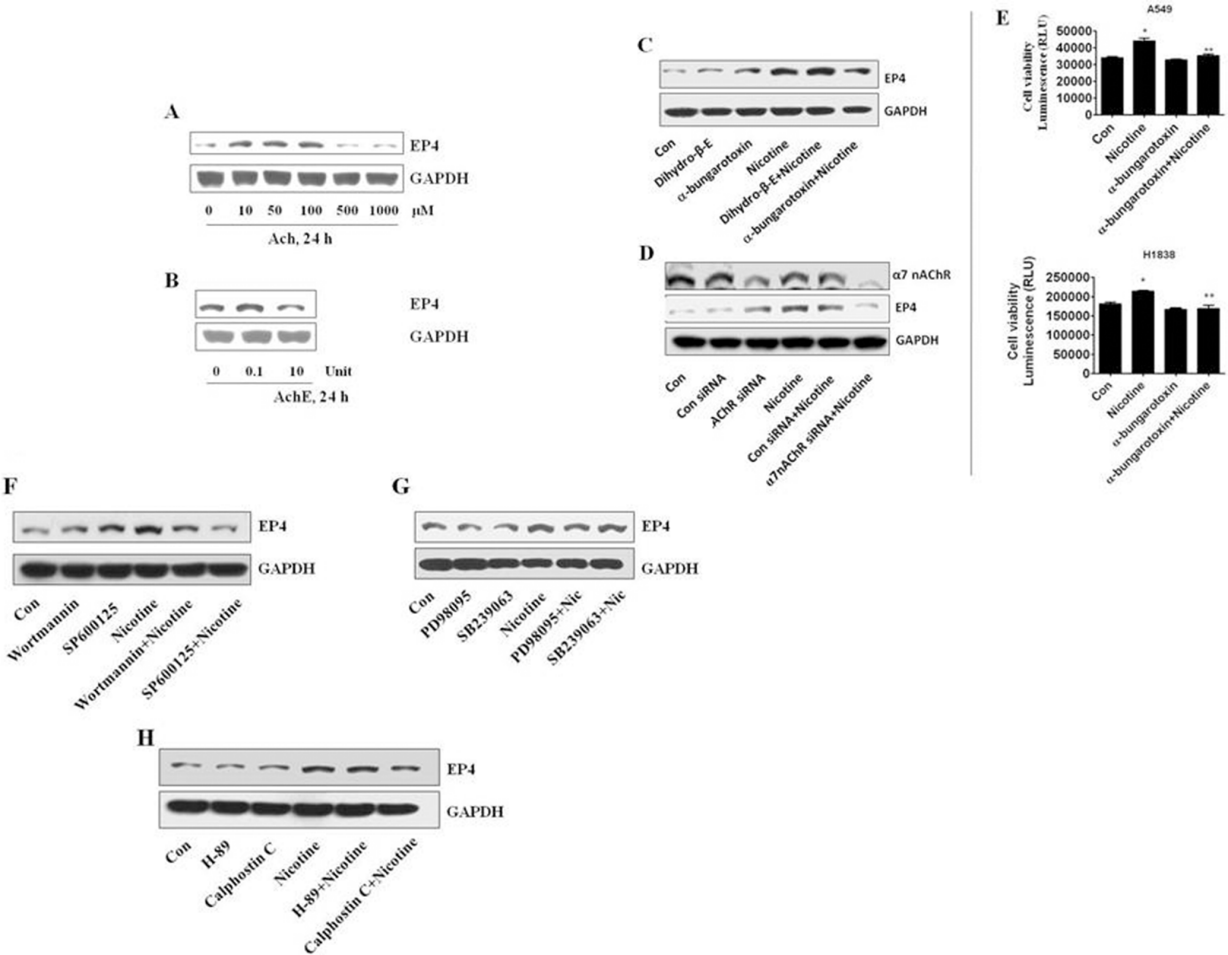
Involvements of α7 nAChR, and PI3K, JNK and PKC pathways in the induction of EP4 by nicotine (**A**) Acetylcholine increased the expression of EP4 induced by nicotine in dose-dependent in A549 cells. (**B**) Acetylcholinesterase decreased the expression of EP4 induced by nicotine in dose-dependent in A549 cells. (**C**) a-bungarotoxin of a a7 nAChR blocker, not dihydro-β-erythroidine of a α4 nAChR inhibitor, decreased the expression of EP4 induced by nicotine in A549 cells. (**D**) α7 nAChR siRNA (100 nM) decreased the expression of EP4 induced by nicotine in A549 cells. (**E**) a-bungarotoxin, a a7 nAChR blocker, decreased the proliferation induced by nicotine in A549 cells and H1838 cells. (**F**) The specific inhibitors of PI3-K (wortmannin, 1 μM), JNK (SP600125, 20 μM) reduced expression of EP4 induced by nicotine in A549 cells. (**G**) The specific inhibitors of ERK (PD98095, 20 μM), not P38 MAPK (SB239063, 10 μM) had a minor effect on reduction of EP4 induced by nicotine in A549 cells. (**H**) The specific inhibitors of PKC (calphostin C, 0.5 μM), not PKA (H89, 10 μM) reduced expression of EP4 induced by nicotine in A549 cells. GAPDH served as internal control for normalization purposes.

Since nicotine has been shown to stimulate human lung carcinoma cell proliferation through nicotinic acetylcholine receptor (nAChR)-mediated signals, we assumed that these receptors would be important here. To begin to test this possibility, we exposed the cells to acetylcholine, a natural ligand of nAChRs. We showed that acetylcholine induced EP4 expression in a dose-dependent manner with maximal effect at a concentration of 100mM, compared to the control group (Figure 3A, 1.422 ± 0.201 vs 1.012 ± 0.028, *P* < 0.01). In contrast, and as expected, acetylcholinesterase, which hydrolyzes acetylcholine, reduced EP4 protein expression (Figure 3B, 0.837 ± 0.119 vs 1.010 ± 0.045, *P* < 0.01).

We then tested the role of the α7 nAChR. We showed that a a7 nAChR blocker, a-bungarotoxin, decreased the effect of nicotine on EP4 protein expression in A549 cells (Figure 3C, 1.385 ± 0.194 vs 1.648 ± 0.209, *P* < 0.01). Interestingly, the α4 nAChR inhibitor, dihydro-β-erythroidine, had no effect (Figure 3C, 1.692 ± 0.211 vs 1.648 ± 0.209, *P* > 0.05). We also silenced the expression of the a7 nAChR gene and found that this blocked the stimulatory effect of nicotine on EP4 expression (Figure 3D, 0.963 ± 0.073 vs 1.165 ± 0.092, *P* < 0.05). Note that the α7 nAChR inhibitor also blocked cell proliferation induced by nicotine in A549 and H1838 cells (Figure 3E).

Nicotine has been shown to affect kinase signaling pathways in several studies [9–12] Therefore, we examined the possible role of several kinases in nicotine-induced EP4 expression. We showed that the specific inhibitors of PI3-K (Wortmannin), JNK (SP600125) and PKC (Calphostin C) reduced nicotine-induced EP4 protein expression in A549 cells (Figure 3F and 3H, 1.275 ± 0.147 vs 1.512 ± 0.241, *P* < 0.01; 1.153 ± 0.125 vs 1.512 ± 0.241, *P* < 0.01; 1.293 ± 0.174 vs 1.359 ± 0.227, *P* < 0.01). However, PD98095, an inhibitor of ERK, had a minor effect, while SB239063, an inhibitor of p38MAPK, and H-89, an inhibitor of PKA, had no effect (Figure 3G and 3H, 1.392 ± 0.255 vs 1.432 ± 0.273, *P* < 0.05; 1.417 ± 0.257 vs 1.432 ± 0.273, *P* > 0.05; 1.349 ± 0.209 vs 1.359 ± 0.227, *P* > 0.05).

### Nicotine increases EP4 gene promoter activity

**Figure 4:**
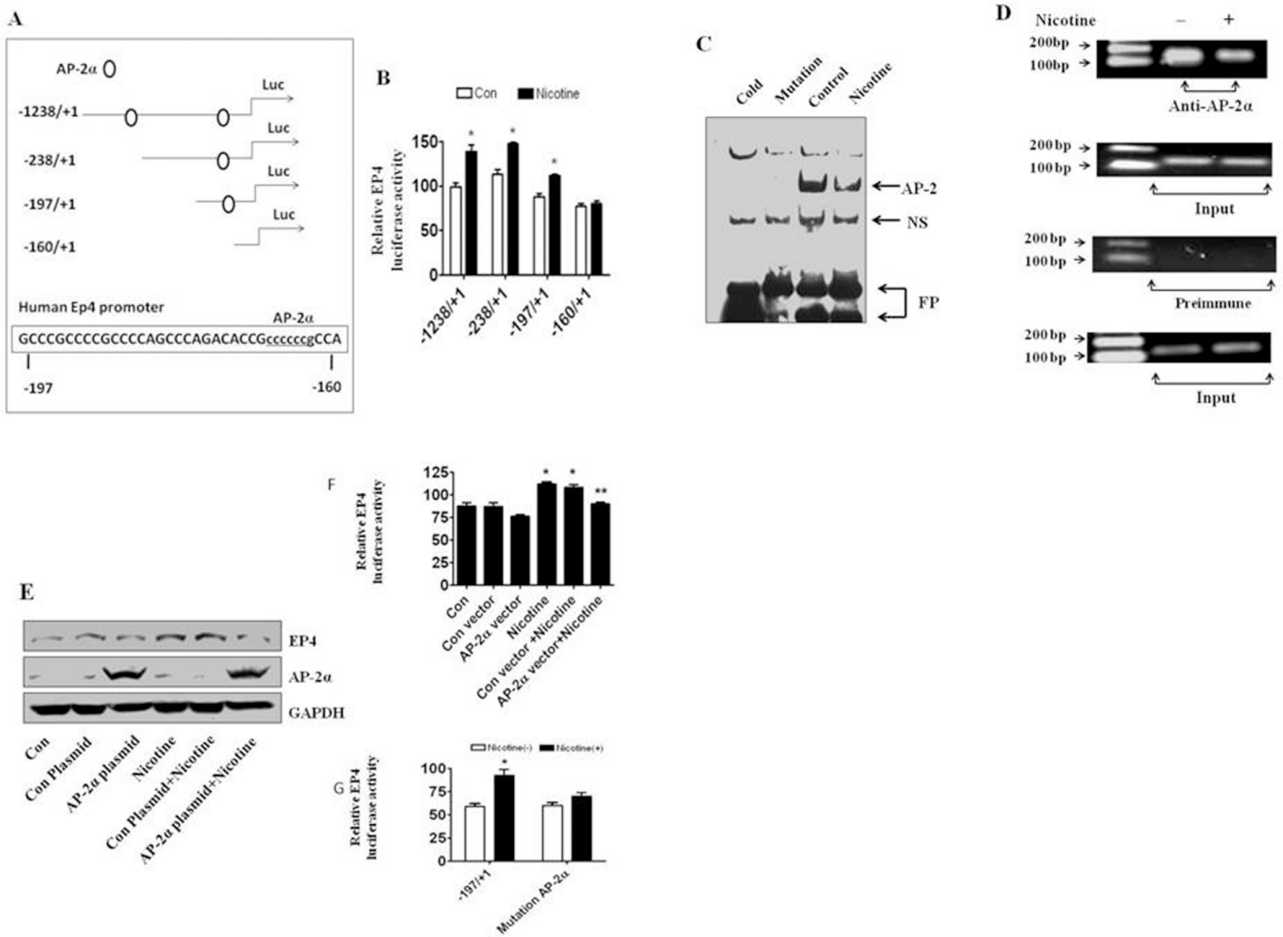
Nicotine stimulates EP4 promoter activity and affects AP-2α binding activity (**A**) The 5′-flanking region of the human *EP4* gene wild type and deletion promoter constructs schematics are presented. These regions contain several transcription factor binding sites including AP-2. (**B**) Nicotine increased EP4 gene promoter activity in A549 cells, transfected with the full-length wild-type EP4 promoter (–1238/+1) luciferase reporter construct and other two EP4 deletion reporter constructs (–238/+1 and –197/+1), not with a shortest EP4 deletion reporter construct (–160/+1). (**C**) Nicotine decreased the binding ability of AP-2α to the Oligonucleotides which contains the AP-2α site. (**D**) Nicotine decreased the EP4 promoter DNA quantity binding to AP-2α protein. (**E**) AP-2α overexpression vector blocked nicotine-induced EP4 protein expression in A549 cells. (**F**) AP-2α overexpression vector blocked nicotine-induced promoter activity of EP4 in A549cells. (**G**) Site-directed mutation of AP-2α (–169 bp ) on EP4 promoter blocked nicotine-induced promoter activity of EP4 in A549cells. ^*^indicates significance as compared with controls. ^**^indicates significance of combination treatment as compared with nicotine alone (*P* < 0.05). *Con*, untreated control cells.

We next examined whether the effects of nicotine on EP4 expression occur at the transcriptional level. The EP4 promoter contains multiple transcription factor binding sites including AP-2*α, among others* (Figure 4A). This site has been shown to be differentially responsive to various stimuli [13–15]. We found that A549 cells, transfected with the full-length wild-type EP4 promoter (–1238/+1) luciferase reporter construct, showed increased promoter activity in response to nicotine exposure (Figure 4B). The nicotine-induced EP4 promoter activity was also observed in two other EP4 deletion reporter constructs (–238/+1 and -197/+1). However, there was no response to nicotine with a shortest EP4 deletion reporter construct (–160/+1) (Figure 4B). These results suggested that the region between -197 and -160 of the EP4 gene promoter played an important role in mediating the effect of nicotine on EP4 gene expression. To further explore the role of nicotine in regulation of EP4 gene promoter activity, EMSA assays were performed to identify the transcription factors involved. We found that A549 cells treated with nicotine for 24 h showed a significant decrease in AP-2α/DNA binding activity (Figure 4C). Furthermore, ChIP assays showed a reduction in AP-2α induced by nicotine binding to specific DNA sequences in the *EP4* gene promoter (Figure 4D).

### Role of AP-2α in EP4 gene expression by nicotine in human lung carcinoma cells

Considering the data presented above implicating AP-2α, We found that overexpression of AP-2α blocked nicotine-induced EP4 protein expression (Figure 4E, 1.116 ± 0.098 vs 1.257 ± 0.134, *P* < 0.01) and promoter activity (Figure 4F). The control plasmid had no effect. Site-directed mutagenesis analysis showed that the stimulatory effect of nicotine on EP4 gene promoter activity was not observed with one EP4 promoter construct in which one AP-2α site (–169 bp) (see Figure 4A) was mutated (Figure 4G).

## DISCUSSION

Although nicotine is not a carcinogen by itself, it has been shown to induce tumor cell proliferation and differentiation [16]. The mitogenic effects of nicotine in NSCLC are analogous to those of growth factors and involve activation of multiple signaling pathways [17]. nAChRs seem to play an important role in mediating the effects of nicotine on cell proliferation and survival [17]. Nicotine up-regulates α7 nAChR expression in NSCLC cells, which could amplify the effects of nicotine [18, 19]. In this study, we didn't see elevation of α7 nAChR expression. But silencing α7 nAChR by siRNA or inhibition of α7 nAChR by its inhibitor could block the effect of nicotine on EP4 whereas α4 nAChR inhibitor has no effect, which means nicotine could induce EP4 expression by activation of α7 nAChR.

Herein, we show that nicotine can also induce NSCLC cell proliferation by triggering the release of PGE_2_ and activating EP4 receptors. In fact, silencing of EP4 inhibited the mitogenic effect of nicotine. EP4 overexpression has been demonstrated in many cancer cells including lung carcinoma cells, and has been shown to be involved in promoting the growth and invasion of NSCLC in many experimental systems [20] and poor prognosis in patients [21].

The effects of nicotine on cell proliferation and on EP4 expression were mediated via nAChRs as demonstrated by the fact that acetylcholine, an endogenous nAChR ligand, mimicked the effect of nicotine. Furthermore, acetylcholinesterase, which degrades acetylcholine, reduced the expression of EP4. Furthermore, we showed that α7 nAChR, but not α4 nAChR, mediated the effect of nicotine on EP4 expression. This is consistent with work showing that nicotine stimulates NSCLC cell survival through muscle-type and neuronal nAChRs suggesting that endogenous acetylcholine released locally in the lung and/or chronic nicotine exposure might play a role in NSCLC progression [22].

We also studied the signaling pathways responsible for EP4 expression in response to nicotine. Data from our laboratory and that of others have demonstrated that nicotine activates several kinase signaling pathways including ERK, JNK, PI3K, PKC, and PKA [22–25]. Here, we report that inhibitors of PI3-K, JNK and PKC reduced nicotine-induced EP4 protein expression. In contrast, ERK, p38MAPK, and PKA played an insignificant role in the up-regulation of the *EP4* gene induced by nicotine. In other work, ERK and p38MAPK was implicated in the effects of troglitazone or curcumin on glioblastoma or H&N cells [13, 14] suggesting the existence of independent pathways that differ according to the stimulus.

**Figure 5:**
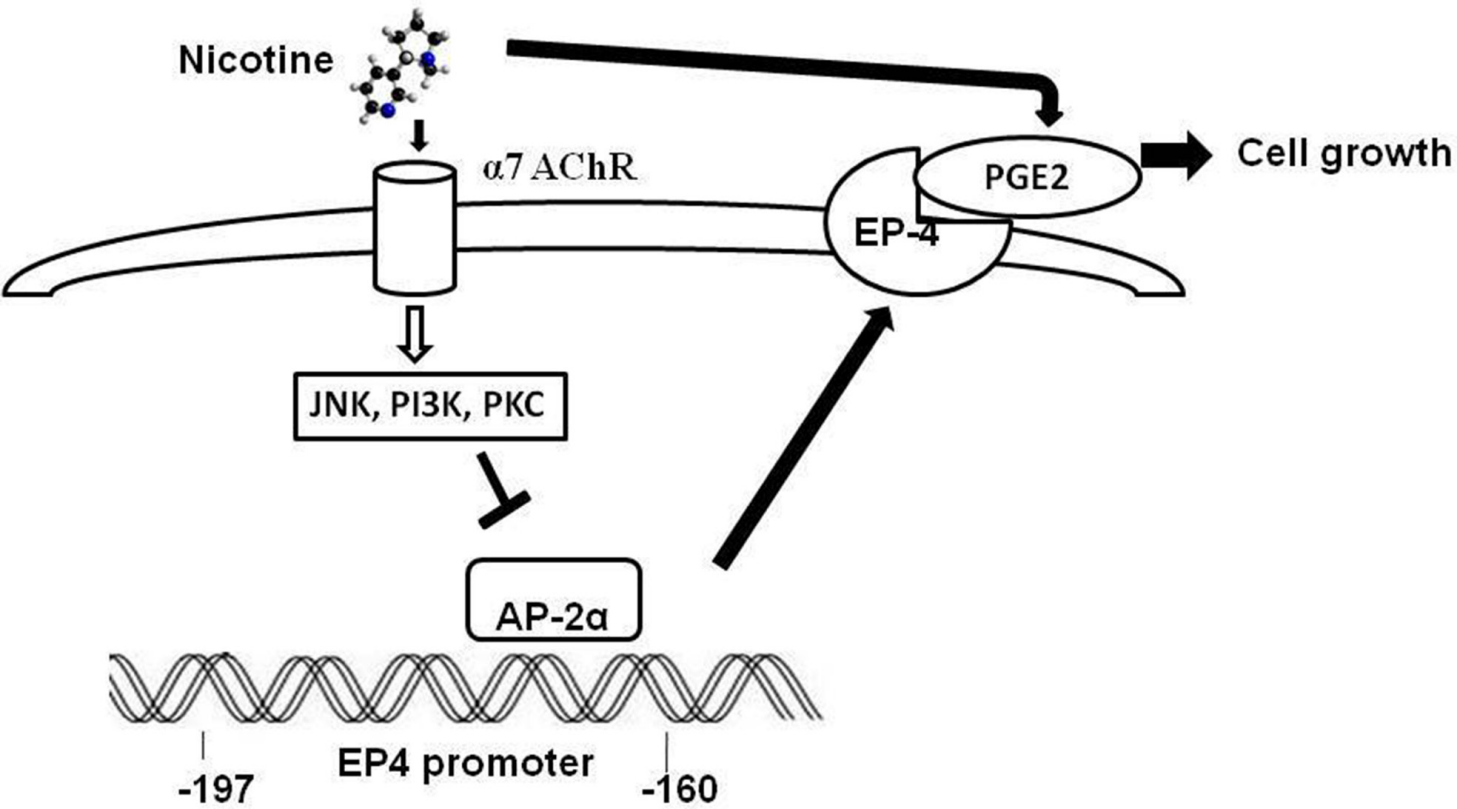
The novel mechanism of nicotine increasing EP4 expression and NSCLC proliferation

EP4 is regulated at the level of gene transcription in different cell types [13, 26]. We found that nicotine increased human *EP4* promoter activity in the –1238/+1, –238/+1 and 197/+1, but not in the –160/+1 DNA constructs; the region between –197 and –160 appeared to play a critical role. Several transcription factor binding sites within regions of the *EP4* gene promoter have been characterized, including regulatory elements for AP-2, C/EBP, Sp1, and others. We showed that treatment of A549 cells with nicotine significantly decreased AP-2α protein binding to specific DNA sequences in the *EP4* gene promoter. Overexpression of AP-2α can alleviates the EP4 induction by nicotine. AP-2α participates in the regulation of important cellular processes including apoptosis, cell growth, and cell differentiation [27]. Down-regulation of AP-2α in tumor cells has been reported in melanoma and in breast, prostate, lung, and colon cancers suggesting that the loss of AP-2α is associated with a malignant phenotype [27]. Furthermore, overexpression of AP-2α has been shown to suppress tumorigenicity suggesting that AP-2 α may function as a tumor suppressor gene [28]. Others have reported that overexpression of AP-2α inhibits breast cancer cell growth by inducing the expression of the cell cycle inhibitor p21, repressing cyclin D1, decreasing Rb phosphorylation, and by enhancing E2F activity [29]. In this experiment, the results strongly suggest that AP-2α play a crucial role mediates nicotine induced EP4 expression by decreased binding ability to EP4 promotor (Figure 5).

## MATERIALS AND METHODS

### Culture and chemicals

The human NSCLC cell lines A549 and H1838 were obtained from the American Type Culture Collection and cultured in RPMI 1640 with 10% heat-inactivated fetal bovine serum as previously described. Cells were plated into six-well culture plates at an initial seeding density of 5 × 10^4^ cells per well. The plates were incubated in a humidified atmosphere of 5% CO_2_ in air at 37°C. Lipofectamine 2000 reagent was purchased from Invitrogen (Carlsbad, CA). The CellTiter-Glo Luminescent Cell Viability Assay kit, Gel Shift Assay System and the Dual-Luciferase Reporter Assay kit were obtained from Promega (Madison, WI). The ERK inhibitor PD98059 was purchased from Cell Signaling (Beverly, MA). The EP4 inhibitor (AH23848) and the EP4 receptor (C-Term) polyclonal antibody were purchased from Cayman Chemical Co (Ann Arbor, Michigan). Polyclonal anti-α7nAChR was purchased from Abcam (Cambridge, MA). The α7 nAChR (sc-42532), EP4 (sc-40173) and control (sc-37007) siRNAs, and polyclonal antibodies against AP-2α were purchased from Santa Cruz Biotechnology (Santa Cruz, CA). The p38 MAPK inhibitor SB239063, the α7 nAChR inhibitor α-bungarotoxin, the α4 nAChR inhibitor dihydro-β-erythroidine, the PI3K inhibitor wortmannin, the PKC inhibitor calphostin C, the PKA inhibitor H89, Acetylcholine (Ach), and acetylcholinesterase were purchased from Sigma Aldrich (St. Louis, MO).

### Western blot analysis

Cells were washed and lysed in 0.15 ml cell extraction buffer (Invitrogen). Equivalent amounts of protein were solubilized in 2× SDS sample buffer, separated on 10% SDS-polyacrylamide gels, transferred onto nitrocellulose membrane, blocked with 5% nonfat dry milk containing 0.1% Tween 20 for 1 h at room temperature, and washed thrice with wash buffer (1×TBST). Blots were incubated with primary antibodies against AP-2α, α7 nAChR at 1:1000 dilution, or EP4 (1:4000), overnight at 4°C, then washed thoroughly, and incubated with secondary anti-rabbit IgG conjugated to horseradish peroxidase (1:2,000 dilution; Santa Cruz) for 1 h at room temperature. Blots were stained by ECL regents (Amersham Life Science) and exposed to X-ray film, and proteins were quantified by densitometric scanning using a Bio-Rad GS-800 calibrated densitometer.

### Reverse transcription and real time PCR

Real time PCR was performed to assess whether EP4 expression was modulated by nicotine. Total RNA was isolated from the cells exposed to nicotine using RNA-Bee RNA isolation reagent (Arms Biotechnology) according to the manufacturer's instructions. Real-time RT-PCR reactions were performed using PerfeCTa SYBR. To amplify the EP4 and GAPDH cDNA fragments, the samples were processed using a Cepheid Smart Cycler: denatured at 95°C for 120 s, followed by 40 cycles, each with temperature variations as follows: 95°C for 1 s, 60°C for 30 s. Results of the log-linear phase of the growth curve were analyzed and relative quantification was performed using the 2^–ΔΔ^CT method. Gene expression of PDK1 is expressed relative to GAPDH and untreated samples in each stimulation study, respectively. At least 3 replicates were run for each condition.

### Short interfering RNA treatment

Cells (70% confluence) were transfected with EP4, α7 nAChR or control siRNAs using Lipofectamine 2000 reagent. Briefly, Lipofectamine 2000 was mixed and incubated with Opti-MEM medium for 5 min, then mixed with siRNA (100 nM), incubated for 20 min at room temperature before the mixture was added to cells. After culturing for 48 h in DMEM medium with 0.5% FBS, cells were treated with or without nicotine for an additional 24 h, and analyzed by Western blot or cell viability assay.

### Transient transfection assays

Human EP4 promoter wild, deletion and mutation constructs ligated to a luciferase reporter gene (PGL3-Basic) have been reported previously [13]. Briefly, NSCLC cells (5 × 10^5^ cell / well, 50–60% confluence) were transfected with EP4 promoter plasmids or EP4 promoter mutation plasmids DNA (2 μg/well) and internal control pRL-CMV Renilla luciferase reporter DNA (0.02 μg/well) using Lipofectamine 2000 reagent as previously described. Cells were transfected with control plasmid (2 μg/well) or with AP-2α expression plasmid (1 μg/well) SP(RSV)AP-2 (#12100) (purchased from Addgene, Inc.; Cambridge, MA) [30] and treated with or without nicotine (0.5 μM) for an additional 24 h before luciferase activity was determined using the Dual-Luciferase Reporter kit (Promega). Firefly luciferase activity was normalized with Renilla luciferase activity within each sample.

### ELISA assay

ELISA assay was performed using the prostaglandin E_2_ EIA kit (Cayman) according to the manufacturer's protocol. Briefly, A549 cells (3 × 10^6^) were treated with the indicated conditions for 24 h. Cell culture supernatants were collected and diluted in EIA buffer. Plates were prepared as described in the manufacturer's protocol and were incubated for 18 h at 4 C. Afterwards, the wells of plates were washed and rinsed 5 times with washing buffer, and the Ellmans reagent and 5 μL tracer were added and incubated in dark for 90 min followed by reading the plate at a wavelength of 405 and 420 nm.

### Cell viability assay

NSCLC cells were treated with the EP4 inhibitor, AH23848, or the α7 nAChR inhibitor, α-bungarotoxin, for 2 h or were transfected with control or EP4 siRNA for 48 h before exposure of the cells to nicotine (0.5 μM) for an additional 72 h in 96-well plates in DMEM media with 0.5% FBS. Afterwards, cell viability was measured using the CellTiter-Glo Luminescent Cell Viability Assay kit (Promega) according to the instructions of the manufacturer.

### Site-directed mutagenesis

EP4 constructs incorporating point mutations in the AP-2α binding site were created using Quikchange II site-directed mutagenesis kit (Stratagene, La Jolla, CA) according to the manufacturer's protocol. The Ap-2α binding site was point-mutated to the two TT base pairs (indicated by underline) in the EP-4(-197/+1) constructs, and primer designed were as follow: Mut AP-2: 5′- CCAGAC ACCGCCCCTTGCCAGTCTTCCCTGC-3′. The sequence of each construct was verified to confirm the incorporation of the appropriate mutation. The EP4 promoter constructs (–1238/+1, –238/+1, –197/+1, –160/+1) and SP-1 mutation constructs (Sp1A, Sp1B, Sp1A1B) were gift from Thomas E. Eling (Laboratory of Molecular Carcinogenesis, National Institute of Environmental Health Science, National Institutes of Health).

### Electrophoretic mobility shift assays (EMSA)

EMSA experiments were performed using Lightshift Chemiluminescent EMSA Kit (Pierce) according to the manufacturer's instructions. Nuclear protein extracts from NSCLC cells treated with nicotine were prepared for EMSA. The single-stranded oligonucleotides for wild type AP-2 were as follows: 5′-GATCGAACTGACCGCCC GCGGCCCGT-, Mutation primer was 5′-GATCGAACTG ACCGCTTGCGGCCCGT-3′. Briefly, nuclear proteins (5 μg) from control and nicotine-treated cells were incubated with annealed double-stranded biotin end-labeled oligonucleotide probe (20 fmol) at room temperature for 20 min. For cold competition, a 200-fold excess of the respective unlabeled consensus oligonucleotide was added before adding probe. Samples were separated on a native 6% polyacrylamide gel and then transferred to a nylon membrane. Subsequently, the membrane was cross-linked, blocked and incubated in conjugate/blocking buffer. After 4 times of washing, the membrane was equilibrated, transferred to the substrate working solution for 5 min and exposed to X-ray film for 20 min.

### Chromatin immunoprecipitation (ChIP) assay

ChIP assay was performed using the ChIP assay kit (Upstate Biotechnology) according to the manufacturer's protocol. Briefly, A549 cells (1 × 10^6^) were treated in the indicated conditions for 24 h and then fixed with 1% formaldehyde for 10 min at 37°C. The fixed cells were scraped into conical tubes, pelleted, and lysed in SDS lysis buffer containing 1 mM phenylmethylsulfonyl fluoride, 1 μg/ml aprotinin, and 1 μg/ml pepstatin A. DNA was sheared to fragments of 200–800 bp by sonication 8 times for 5 s. The sonicated cell supernatant was diluted 10 fold in the ChIP dilution buffer and 1% of the diluted cell supernatant was kept as a positive control (Input). The chromatin was precleared with salmon sperm DNA/protein A-agarose slurry for 1 h at 4°C. The precleared supernatant was incubated with antibodies against AP-2α (Santa Cruz) or normal rabbit IgG overnight at 4°C. The immunocomplexes were eluted with elution buffer (1% SDS, 0.1 M NaHCO_3_, 10 mM DTT). NaCl (5M) was added into eluted samples to reverse histone-DNA crosslinks and the samples were heated overnight at 65°C. Purified DNA samples were used as a template for PCR amplification. The region between −238 and −103 of the human EP4 promoter was amplified using the following primers: 5′-CTCCGAGGGCGTGAAAAC-3′ (sense), 5′-CATTGGCCGGATTGGAAG-3′ (antisense). The 136 bp products were resolved on a 1% agarose gel and visualized under UV light.

### Statistical analysis

All experiments were repeated a minimum of three times. All data from western blot analysis, real-time PCR, luciferase assays are expressed as mean ± SD. The data presented in some figures are from a representative experiment, which was qualitatively similar in the replicate experiments. In cell viability assay, the bar graphs represented the mean ± s.d. of relative cell viability compared to the control group of at least three independent experiments. In western blot analysis, the optical densities (OD) of EP4 and AP-2a were normalized to the OD of GAPDH in the same membrane. The data represented the mean ± s.d. of relative OD compared to the control group of at least three independent experiments with 3 samples in each. In transient transfection assay, the bar graphs represent the mean ± s.d. of relative luciferase activities compared to the control group of at least three independent experiments. One-way anova analyses followed by post hoc testing were performed. Asterisks showed in the figures indicate significant differences of experimental groups in comparison with the corresponding control condition. *P*-values < 0.05 were considered statistically significant.

## CONCLUSIONS

In summary, our study shows that nicotine stimulates NSCLC cell proliferation by acting on α7 nAChR and triggering the release of PGE_2_ which, in turn, activates EP4. We also showed that these events might be amplified by the ability of nicotine to stimulate the expression of EP4 through the activation of several kinase signaling pathways. Importantly, nicotine-induced EP4 gene expression is dependent on inhibition of AP-2α binding to the *EP4* gene promoter. To our knowledge, this represents the first demonstration of a link between nicotine and the *EP4* gene, thereby unveiling a novel mechanism by which nicotine stimulates NSCLC cell growth. Targeting downstream molecules that link cholinergic and prostanoid signaling may be an effective strategy against lung cancer.

## Abbreviations

NSCLC: non small lung cancer cell
nAChR: nicotinic acetylcholine receptor
PGE_2_: prostaglandin E2
COX-2: Cyclooxygenases-2
